# Treatment Planning and Delivery of Whole Brain Irradiation with Hippocampal Avoidance in Rats

**DOI:** 10.1371/journal.pone.0143208

**Published:** 2015-12-04

**Authors:** C. K. Cramer, S. W. Yoon, M. Reinsvold, K. M. Joo, H. Norris, R. C. Hood, J. D. Adamson, R. C. Klein, D. G. Kirsch, M. Oldham

**Affiliations:** 1 Department of Radiation Oncology, Duke University Medical Center, Durham, NC, United States of America; 2 Department of Psychiatry, Durham VA, Durham, NC, United States of America; 3 Department of Pharmacology & Cancer Biology, Duke University Medical Center, Durham, NC, United States of America; 4 Department of Anatomy and Cell Biology, Sungkyunkwan University School of Medicine, Seoul, South Korea; 5 Department of Psychiatry and Behavioral Sciences, Duke University Medical Center, Durham, NC, United States of America; Northwestern University Feinberg School of Medicine, UNITED STATES

## Abstract

**Background:**

Despite the clinical benefit of whole brain radiotherapy (WBRT), patients and physicians are concerned by the long-term impact on cognitive functioning. Many studies investigating the molecular and cellular impact of WBRT have used rodent models. However, there has not been a rodent protocol comparable to the recently reported Radiation Therapy Oncology Group (RTOG) protocol for WBRT with hippocampal avoidance (HA) which is intended to spare cognitive function. The aim of this study was to develop a hippocampal-sparing WBRT protocol in Wistar rats.

**Methods:**

The technical and clinical challenges encountered in hippocampal sparing during rat WBRT are substantial. Three key challenges were identified: hippocampal localization, treatment planning, and treatment localization. Hippocampal localization was achieved with sophisticated imaging techniques requiring deformable registration of a rat MRI atlas with a high resolution MRI followed by fusion via rigid registration to a CBCT. Treatment planning employed a Monte Carlo dose calculation in SmART-Plan and creation of 0.5cm thick lead blocks custom-shaped to match DRR projections. Treatment localization necessitated the on-board image-guidance capability of the XRAD C225Cx micro-CT/micro-irradiator (Precision X-Ray). Treatment was accomplished with opposed lateral fields with 225 KVp X-rays at a current of 13mA filtered through 0.3mm of copper using a 40x40mm square collimator and the lead blocks. A single fraction of 4Gy was delivered (2Gy per lateral field) with a 41 second beam on time per field at a dose rate of 304.5 cGy/min. Dosimetric verification of hippocampal sparing was performed using radiochromic film. *In vivo* verification of HA was performed after delivery of a single 4Gy fraction either with or without HA using γ-H2Ax staining of tissue sections from the brain to quantify the amount of DNA damage in rats treated with HA, WBRT, or sham-irradiated (negative controls).

**Results:**

The mean dose delivered to radiochromic film beneath the hippocampal block was 0.52Gy compared to 3.93Gy without the block, indicating an 87% reduction in the dose delivered to the hippocampus. This difference was consistent with doses predicted by Monte Carlo dose calculation. The Dose Volume Histogram (DVH) generated via Monte Carlo simulation showed an underdose of the target volume (brain minus hippocampus) with 50% of the target volume receiving 100% of the prescription isodose as a result of the lateral blocking techniques sparing some midline thalamic and subcortical tissue. Staining of brain sections with anti-phospho-Histone H2A.X (reflecting double-strand DNA breaks) demonstrated that this treatment protocol limited radiation dose to the hippocampus in vivo. The mean signal intensity from γ-H2Ax staining in the cortex was not significantly different from the signal intensity in the cortex of rats treated with WBRT (5.40 v. 5.75, P = 0.32). In contrast, the signal intensity in the hippocampus of rats treated with HA was significantly lower than rats treated with WBRT (4.55 v. 6.93, P = 0.012).

**Conclusion:**

Despite the challenges of planning conformal treatments for small volumes in rodents, our dosimetric and *in vivo* data show that WBRT with HA is feasible in rats. This study provides a useful platform for further application and refinement of the technique.

## Introduction

Since the 1950s, whole brain radiotherapy (WBRT) has been used to treat cancer or prevent metastasis in the brain [1]. Despite the tremendous benefit of WBRT in many clinical situations, there are drawbacks—and the impact on cognitive functioning is often the subject of intense physician-patient conversation. Studying neurocognitive decline after whole brain irradiation is tremendously challenging, but the aggregate prospective and retrospective studies indicate that while WBRT prevents neurocognitive decline due to tumor progression, it nevertheless contributes to subtle/early or profound/late neurocognitive decline in verbal learning [2, 3, 4, 5, 6]. In the setting of good tumor control, neurocognitive decline after WBRT is presumably mediated by the effects of radiation on normal neural tissue. The hippocampus has long been recognized as a key anatomic location for memory. And after studies showed that metastases to the limbic circuit are rare, avoiding the hippocampus during WBRT to try to mitigate cognitive side effects was tested in the multicenter phase II RTOG 0933 trial which quantified neurocognitive changes after WBRT with hippocampal avoidance (HA) [7, 8]. The bulk of the literature investigating the molecular and cellular impact on the brain (and specifically the hippocampus) has used rodent models. But to our knowledge, there has not been a rodent protocol comparable to the RTOG protocol to allow for parallel cellular and molecular investigation. The aim of our research was to develop a hippocampal-sparing whole-brain irradiation protocol in Wistar rats.

## Materials and Methods

### Animals and study design

All animal experiments were reviewed and approved by the Duke Institutional Animal Care and Use Committee (IACUC) under protocol number A125-14-05. Wistar rats were purchased from the Charles River Laboratory and housed in pairs. A low dose, bone-contrasted cone beam CT (CBCT) was acquired of a 12-week old (250-300g) Wistar rat skull while fully sedated with 3% isoflurane sedation on a Xrad 225Cx MicroCT irradiator (40kVp, 2.5mA, 1x1 binning, 0.8mm voxels) [9, 10, 11]. This CBCT was registered to an MRI Wistar rat atlas (provided by Al Johnson and Evan Calabrese at the Center for In Vivo Microscopy at Duke) which had been generated on post-natal day 80 Wistar rats using a 7T MRI [12]. Prior work [13] has introduced methods for treatment localization in the brain based on subject-specific diffusion tensor image (DTI) data. However, the consistency in anatomic volume and structure within inbred rat strains allowed for utilization of the MRI atlas in lieu of acquiring individual MRI scans for each treated rat [14]. Radiation treatment planning was performed in smART-Plan employing an opposed lateral technique with custom 0.5cm lead blocks. Dosimetric verification of hippocampal sparing was performed using radiochromic film. *In vivo* verification of hippocampal avoidance was performed using γ-H2Ax staining of brain sections after delivery of a single 4Gy fraction either with or without hippocampal avoidance. We compared the brains of rats treated with hippocampal avoidance, whole brain treatment, and negative controls. Negative controls were sedated, and imaged with fluoroscopy identically to the treatment cohorts but without any beam-on time. Rats used for *in vivo* verification were eight weeks old in order to facilitate decapitation and removal of the brain without tissue damage. Although the hippocampus increases in volume by more than 1000% (roughly 10 μL to 111 μL) from post-natal day (p) 0 to p80, the change in brain volume plateaus around day 40. Therefore percent changes in the hippocampal volume, brain volume and skull volume between weeks 8 and 12 are likely small.

### Image registration

**Fig 1:**
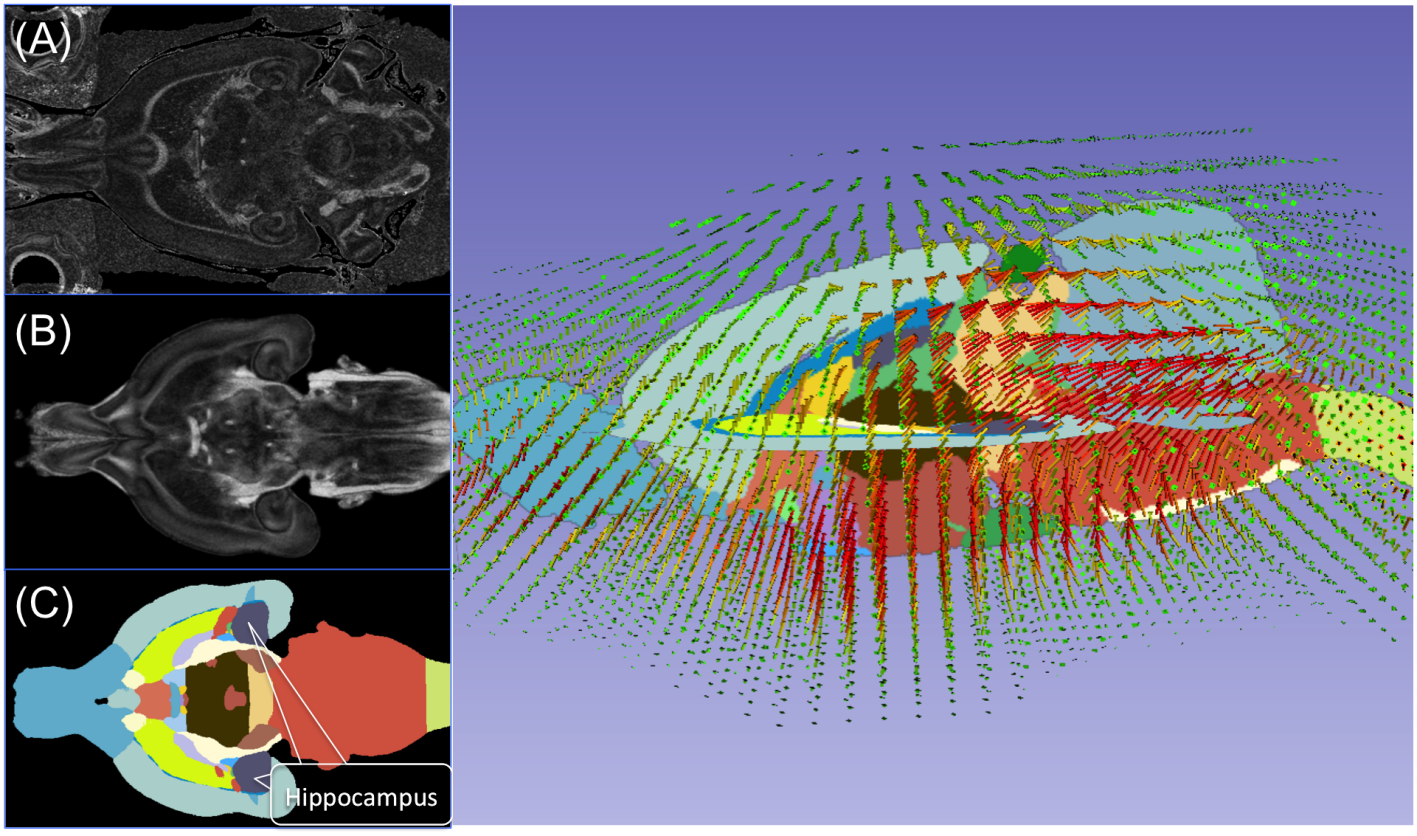
Raw images of (A) the rat brain MRI with skull, (B) atlas, and (C) atlas with anatomic structures segmented and the hippocampus labeled and shown in purple. Once a deformation map was determined from registering the (B) atlas to (A) the skull base MRI based on Mattes mutual information metric using 3D slicer, Version 4.4.0, the deformation map (D) was applied to the atlas segmentation labels (C). Field vectors (on D) are color-coded as such: green arrows represent 0~0.3mm displacement, yellow arrows represent 0.3~0.6mm displacement, and red arrows represent more than 0.6mm displacement. Vector lengths are increased by 50% for better visibility.

**Fig 2:**
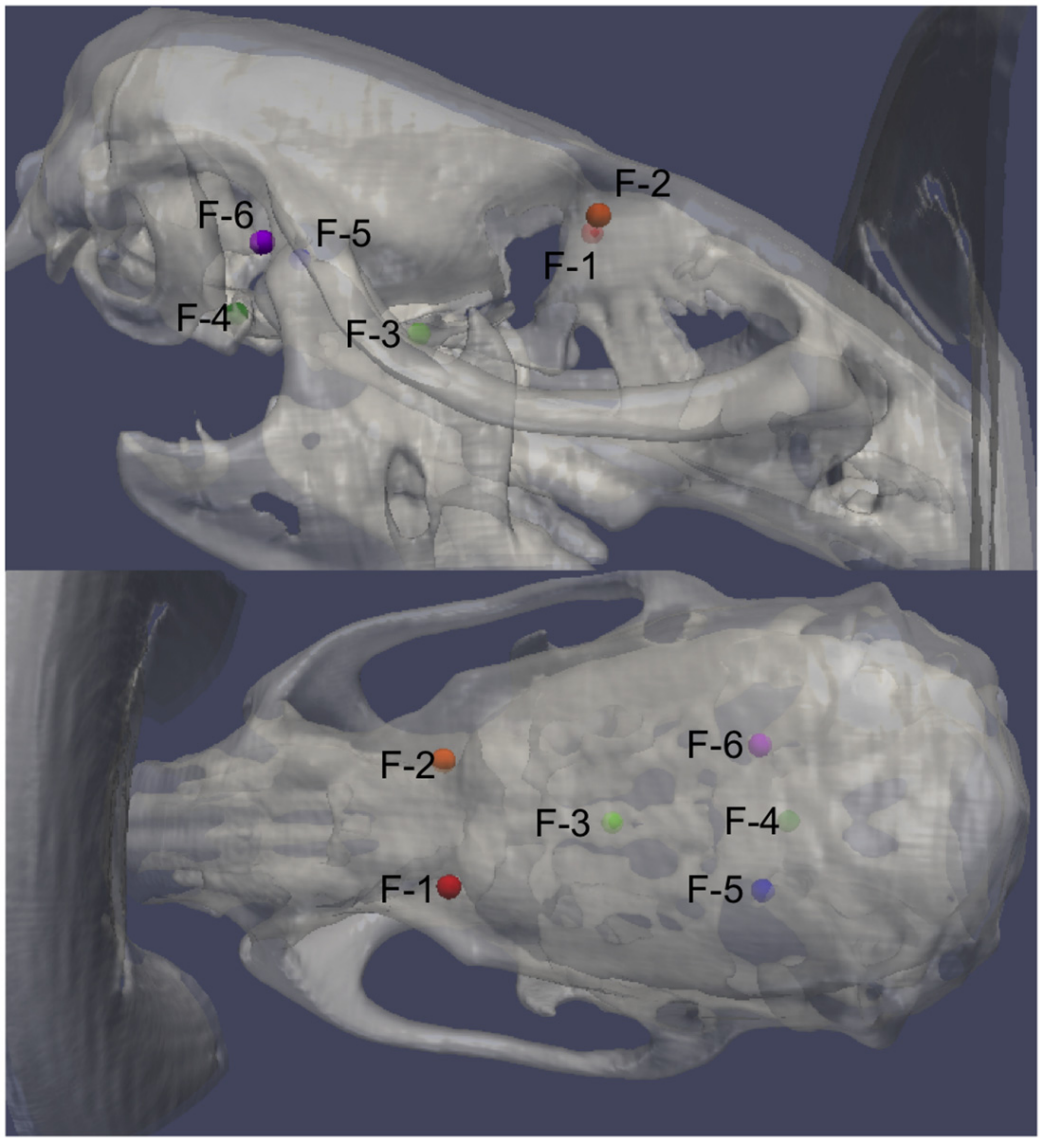
3D view of the six bony landmarks F-1 through F-6 used for registration. Please see S1 Fig and S1 Video for complete CT and MRI details of registration points.

**Fig 3:**
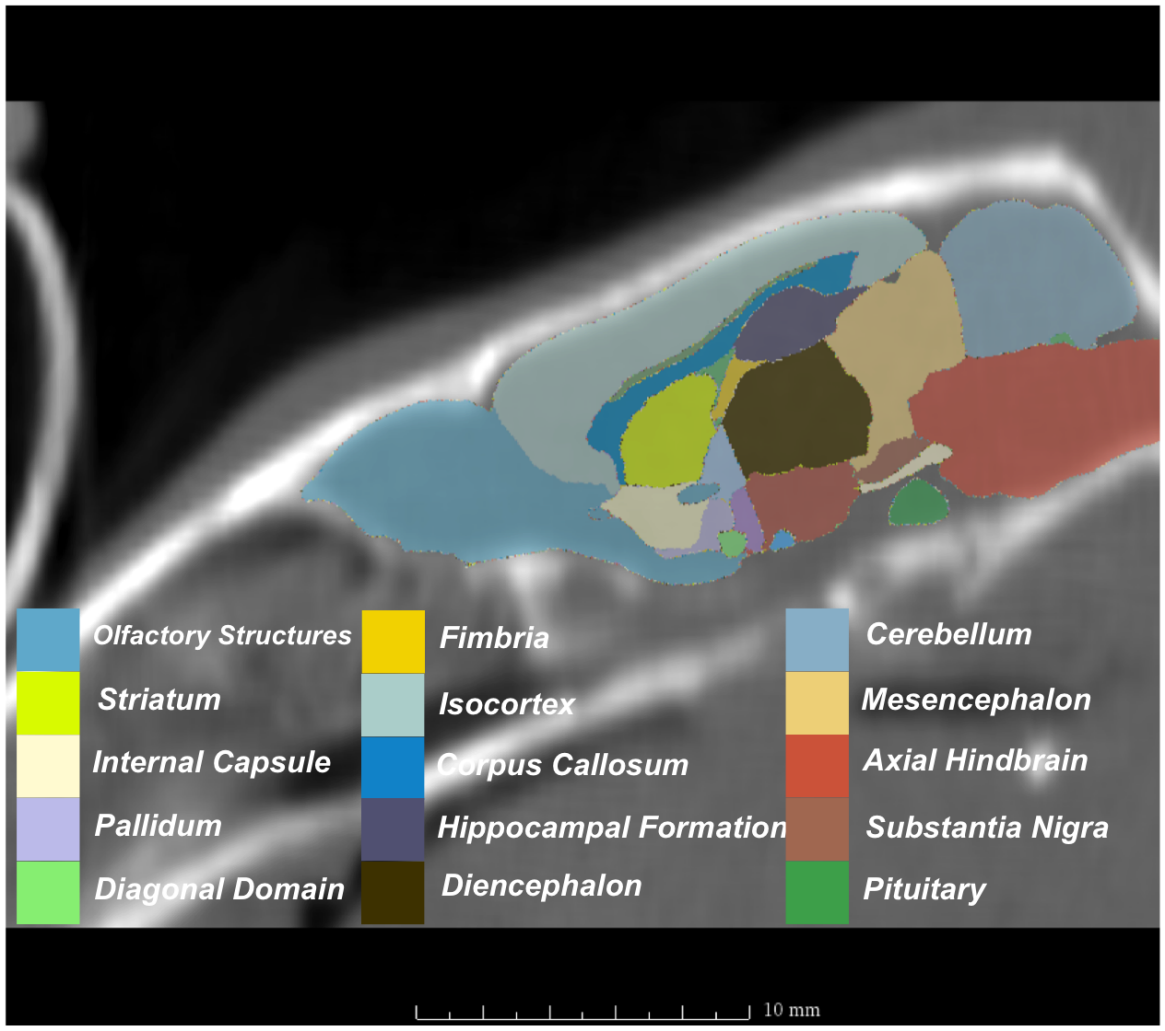
MRI with labeled structures from atlas registered to the CBCT.

**Fig 4:**
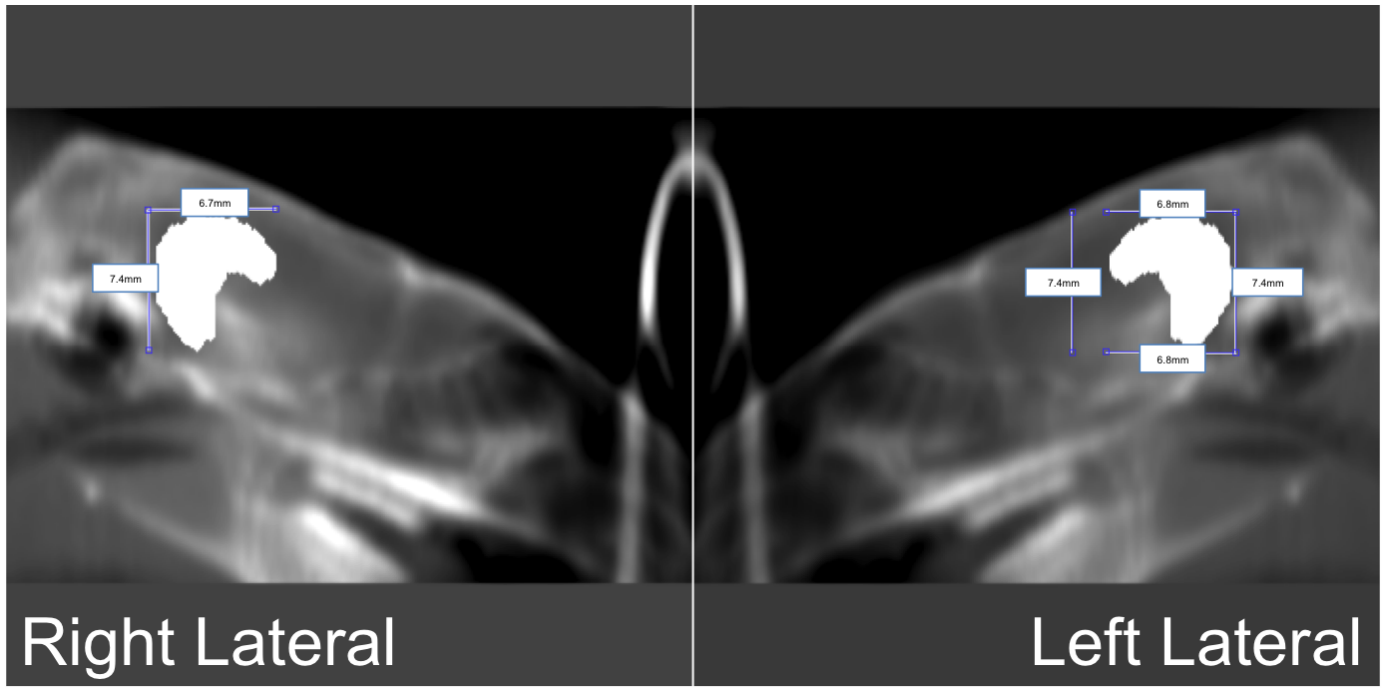
Digitally reconstructed radiographs (DRR) indicating the location of the hippocampus in left lateral and right lateral view of the same rat. The hippocampal block appears hyperintense on the DRR.

Registration was carried out with the open source imaging tool 3DSlicer, Version 4.4.0. Two MRI image sets provided by Duke’s Center for In Vivo Microscopy (CIVM) were initially registered by diffeomorphic B-spline registration—a diffusion tensor image (DTI) MRI with the skull and a labeled (segmented) atlas of the Wistar rat brain. First the General Registration module was used to deformably register the DTI skull-intact MRI (fixed image) to the MRI atlas (moving image) creating a diffeomorphic transform field. This transform field was then applied to the labeled MRI atlas (designated as the “deformed atlas label map”). In essence, this transferred the labels from the atlas to the skull-intact MRI (Fig 1). Subsequently, the skull-intact MRI was rigidly registered to the CBCT using six bony landmarks via the Landmark Registration module available on 3DSlicer. In this registration the CBCT served as the fixed image. Six bony landmarks were identified (Fig 2) to assist in registration. The transform matrix generated from this registration was applied to skull-intact MRI with labels (deformed atlas label map) and the hippocampal volume was isolated. In essence this process used the existing atlas to create a hippocampal contour on the skull-intact DTI MRI and then transferred it to the CBCT (Fig 3) which bypassed the potential for human error in outlining the structure. The CBCT with isolated hippocampal volume was used to generate a digitally reconstructed radiograph (DRR) with Matlab’s iradon function (Fig 4). The DRRs were subsequently used to assist in creating lead blocks and for image guidance during treatment.

### Treatment planning, set up and irradiation

**Fig 5:**
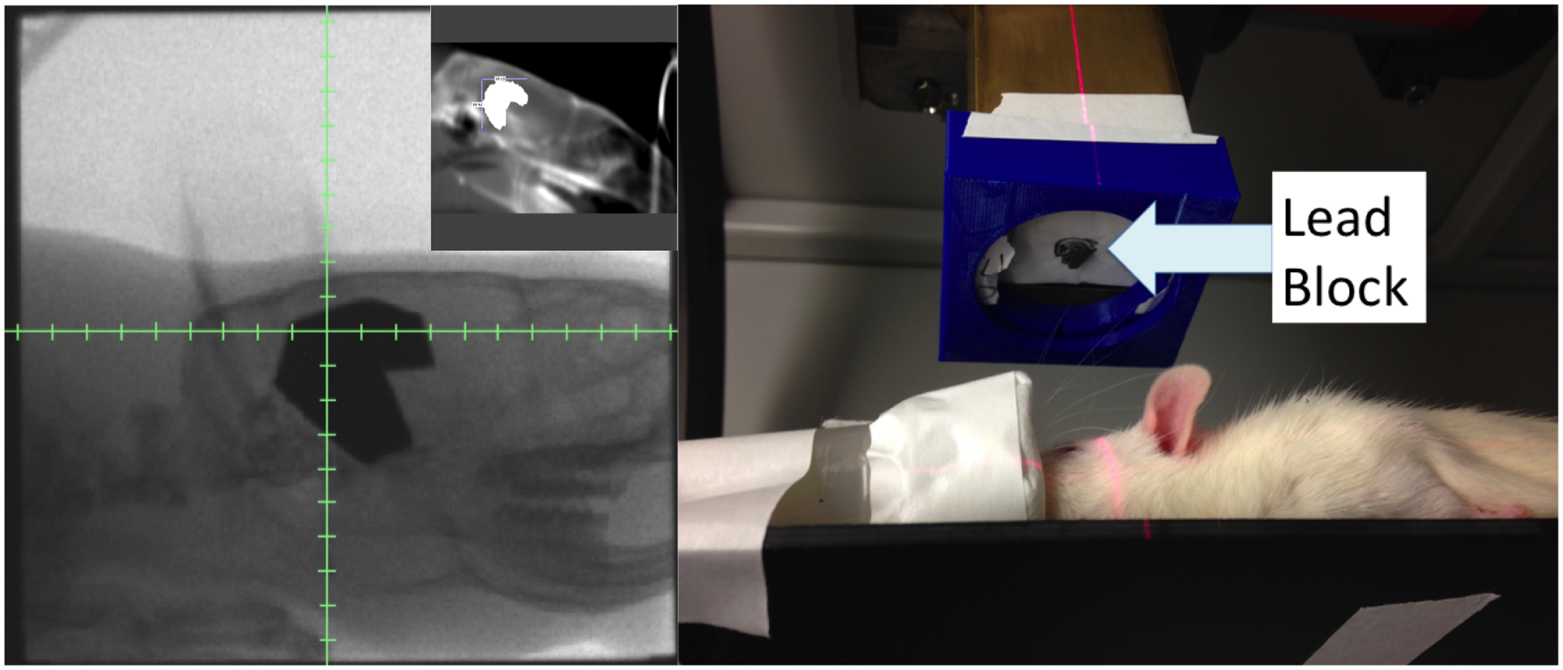
Pictures of actual treatment. Left: Radiograph of the rat with blocks in black. Left inset: Intended hippocampus block on DRR, shown as white region in brain. Right: The blue 3D-printed add-on for the collimator ensures that the treatment is consistent every time it is performed.

Using a representative CBCT from the 8 week old rats, the radiation parameters for the WBRT and HA protocols were generated in SmART-Plan and verified with an independent hand-calculations. Both plans were designed using opposed laterals at gantry angles of 90◦ and 270◦ and a SAD of 30.76cm. The isocenter was positioned at midplane and approximately halfway between the frontal lobe and cerebellum. After ensuring optimal dose coverage and homogeneity, the beam on time and prescription points from smART-Plan were exported to the Xrad 225Cx microCT irradiator (0.5cm below the tissue surface for each beam). Hand calculations were also performed as a second independent check. Blocks for hippocampal avoidance were cut from a 0.5cm-thick lead block, with an intention to leave 1mm margin around the hippocampus. A custom 3D-printed attachment (Fig 5) was created for the collimator end which allowed suspension of the blocks centrally in the collimator, maintained proper orientation of the blocks between treatments and allowed for tiny rotational adjustments of the blocks as needed after fluoroscopic imaging. No attenuation correction was required for the 3D printed attachment as none of it extended beyond the inner edge of the collimator where it could interfere with the treatment beam. Rats were sedated with continuous delivery of 3% isoflurane via a nose cone and positioned prone on the treatment table without immobilization. Several immobilization devices were created. However, they did not easily attach to the stage without causing gantry-collisions. Moreover, the goal of immobilization in the setting of image-guidance during a single treatment is to reduce intra-fraction motion. In a sedated animal this is almost exclusively due to cardiopulmonary motion, which we judged to be small when observing the animals under isoflurane anesthesia by fluoroscopy. After isocenter placement and verification of block positioning through comparison to the digitally-reconstructed-radiograph (DRR) using fluoroscopy, treatments were delivered at maximum energy levels of 225 kVp and 13mA, filtered through 0.3mm of copper, and using a 40x40mm square collimator. 4Gy was delivered from left and right laterals (2Gy per lateral field with a 41 second beam on time per field) at a dose rate of 304.5 cGy/min.

### Dose verification

EBT2 radiochromic film placed between two sets of 0.5cm-thick solid water was irradiated on the X-RAD 225Cx irradiator using two 40mm by 40mm lateral beams. To simulate prescribing a dose of 4Gy to the center of the rat brain with a Source-to-Axis Distance (SAD) set up, the same irradiation protocol as described above was delivered (beam on time of 41 seconds at 225kVp and 13mA resulting in 2Gy per beam to a prescription point of 0.5cm below the tissue surface). Dose verification was done both with and without the hippocampal blocks to evaluate the extent of hippocampal sparing. Furthermore, smART-Plan Monte Carlo simulation was performed on the rat CBCT with originally used to generate the DRRs and lead blocks (simulated lead block structures within the image facilitated the generation of an isodose distribution and DVH accounting for the beam attenuation by the lead blocks).

### In vivo assessment of DNA damage with immunofluorescence

Within one hour of irradiation, three rats receiving whole brain irradiation, three rats receiving the hippocampal avoidance irradiation protocol, and two negative control rats were terminally sedated with CO2, decapitated and their brains removed. The investigator performing the immunofluorescence (KMJ) was blind to the treatment groups. The brains were embedded in clear frozen sectioning compound (VWR, West Chester, PA), frozen on dry ice, and then sliced into 10-μm frozen coronal sections with a cryostat at –20°C. For *immunofluorescence* against phospho-Histone H2A.X, frozen sections were fixed with ice-cold 4% paraformaldehyde for 10 minutes (mins). After permeabilization with PBS containing 0.25% Triton X-100 (PBST) for 10 mins, the sections were blocked by 10% goat serum in PBST for 30 mins at room temperature. Mouse monoclonal anti-phospho-Histone H2A.X (Ser139) Antibody (1:250 in 10% goat serum PBST, #05–636, Milipore, Billerica, MA) and then Alexa Fluor 488-conjuated goat anti-mouse IgG1 (1:400 in 10% goat serum PBST, A-21121, Thermo Fisher Scientific Inc., Waltham, MA) were applied to the sections for 2 and 1 hour, respectively, at room temperature. Nuclei were counter-stained with Hoechst 33342 (1:100 in PBS) for 2 mins at room temperature. To identify neurons selectively, rabbit polyclonal anti-NeuN antibody (1:1000 in 10% goat serum PBST, ABN78, Millipore) with Alexa Flour 555-conjugated goat anti-rabbit IgG antibody (1:400 in 10% goat serum PBST, A-21429, Thermo Fisher Scientific Ins., Waltham, MA) was used. To determine whether differences in the phospho-Histone H2A.X immunoreactivity were statistically significant, we randomly selected an area in the cerebral cortex and hippocampal region (dentate gyrus) of each rat and took pictures of the areas with the same exposure time. Images were acquired with a Leica microscope and signal intensities for phospho-Histone H2A.X were determined using the NIH image program (Image J). Signal intensity is the sum of the green values of all the pixels in the area that was divided by the number of pixels within the area. Average signal intensities were calculated in the cerebral cortex and hippocampal region for each group, which were compared by Student’s t-test.

## Results

### Dose verification

**Fig 6:**
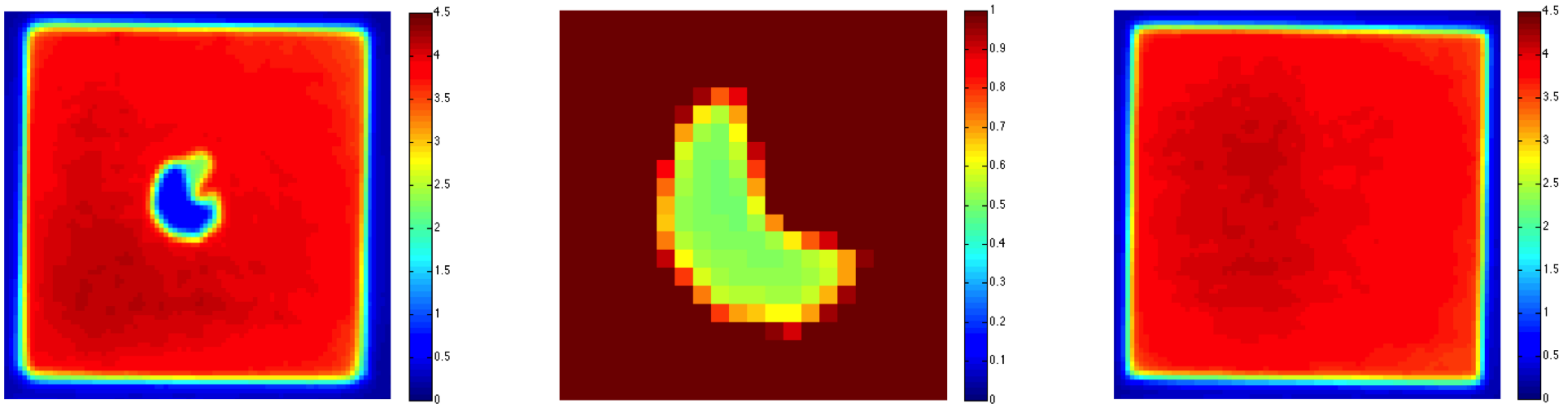
Dose distribution of 4Gy, 40mm by 40mm irradiation (Left) and rescaled (0Gy to 1Gy) dose distribution for the blocked area (Right). Dose of about 0.52Gy to the blocked area can be observed to the right. Without a block, mean dose was 3.93Gy.

**Fig 7:**
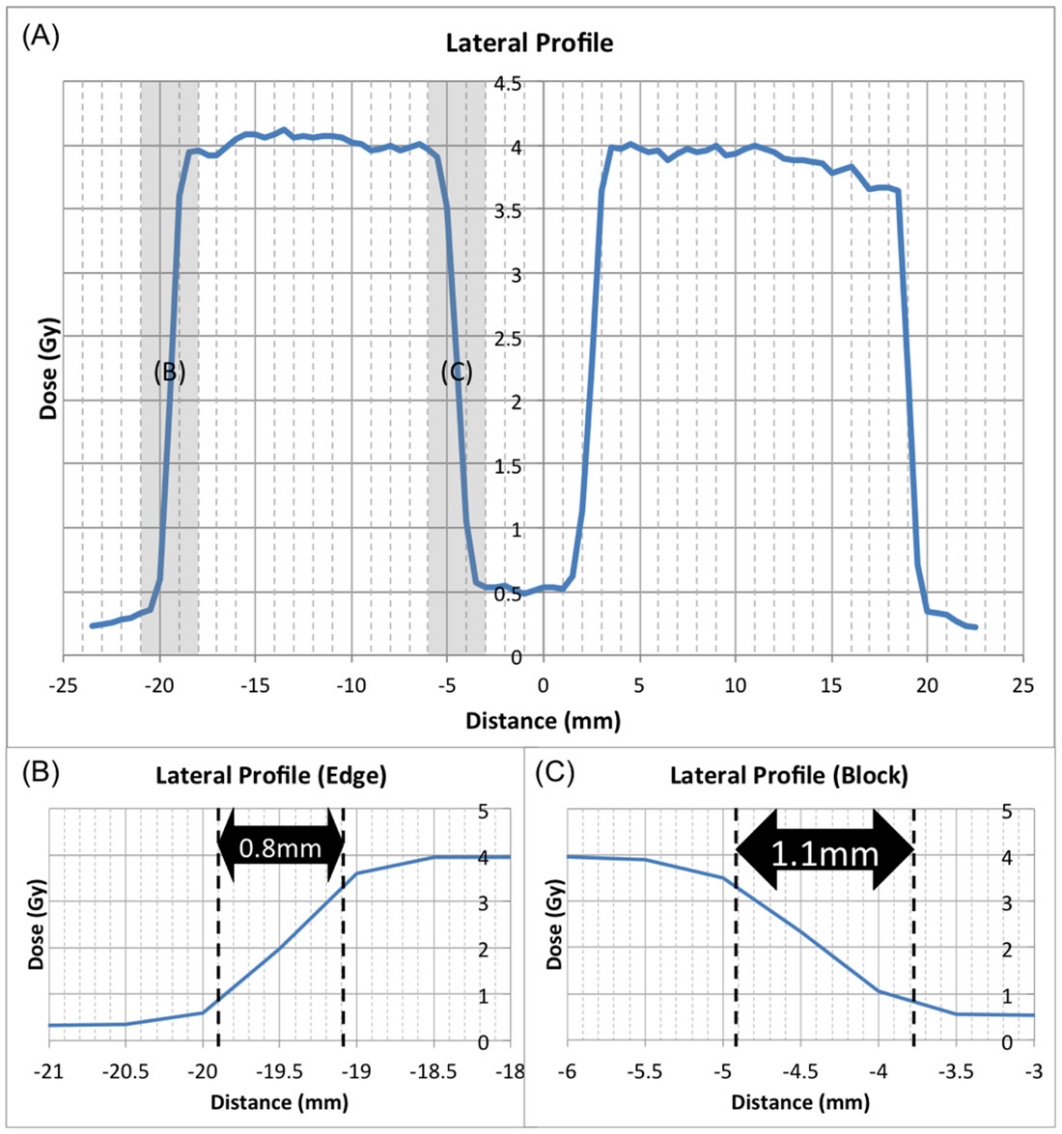
A) Lateral and longitudinal profiles through the block. B) The penumbra (defined as the distance between 80% and 20% of the prescribed dose) measured with radiochromic film at the lateral beam edge (0.8mm) and C) the penumbra at the medial (inner) block edge (1.1mm). No smoothing function or digital processing was applied.

Having designed a hippocampal avoidance treatment plan for rats, we placed radiochromic film as described above and delivered 4 Gy with opposed lateral fields. We measured a mean dose delivered to rachiochromic film beneath the hippocampal block of 0.52Gy (Fig 6 and Table 1). In contrast, when 4Gy was delivered with the WBRT plan without the hippocampal block, we measured a mean dose delivered to the radiochromic film of 3.93Gy. These results indicate that the hippocampal block provided an 87% reduction in the dose delivered to the brain. A dosimetric penumbra (defined as the distance between 80% and 20% of prescribed dose) of 0.8mm was observed at the lateral beam edge while a penumbra of 1.1mm was observed in dose profile generated by the medial (inner) block edge (Fig 7).

**Table 1:** Mean dose, minimum dose, and maximum dose to the blocked and unblocked region of the radiation field.

	Mean Dose	Minimum Dose	Maximum Dose	Standard Deviation
Blocked Region	0.52Gy	0.43Gy	0.60Gy	0.15Gy
Unblocked Region	3.96Gy	3.60Gy	4.37Gy	0.04Gy

**Fig 8:**
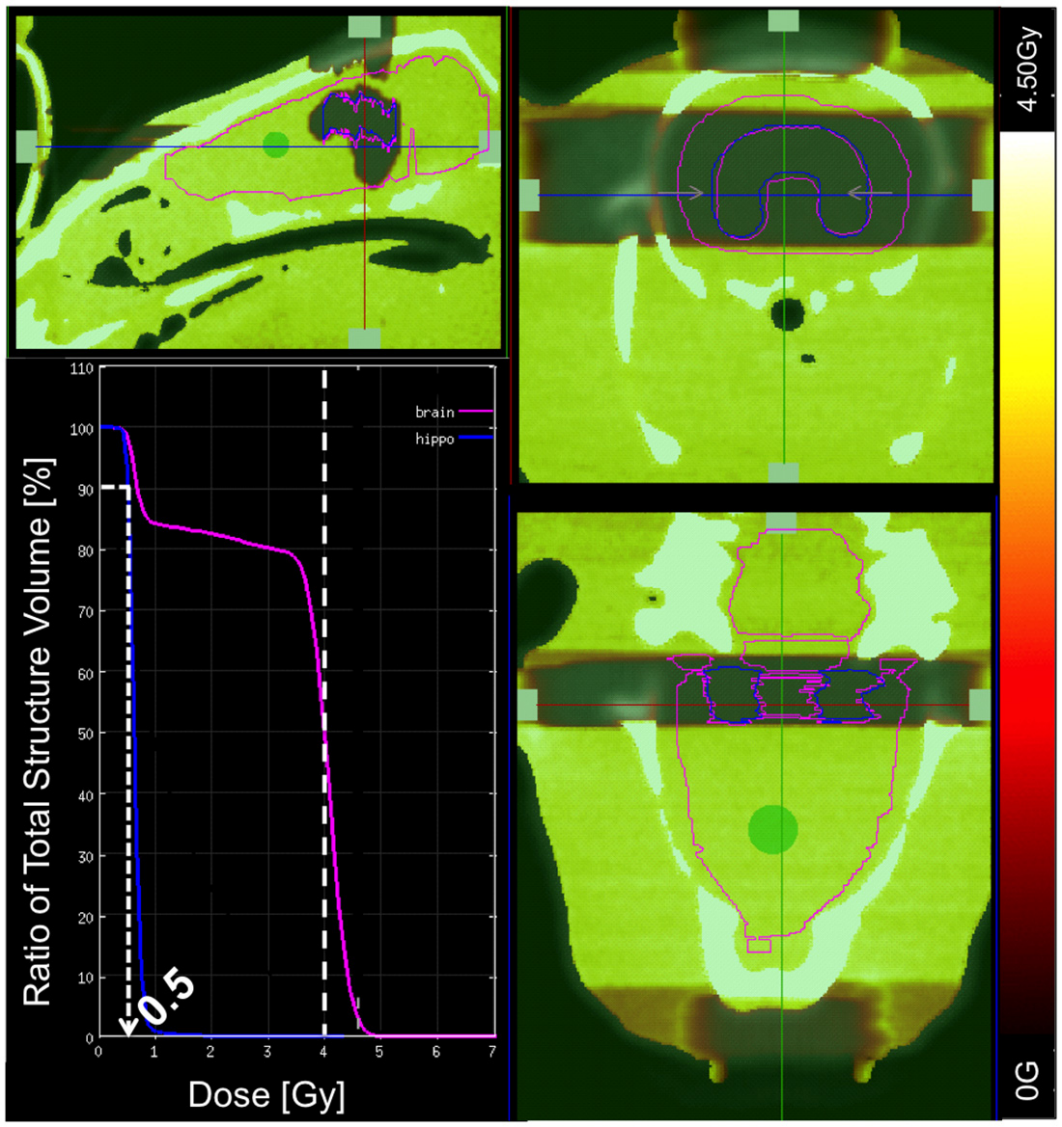
SmART-Plan Dose Volume Histogram (DVH) and dose distribution for simulated irradiation with 100% efficient radiation block. 90% of hippocampus (blue) receives around 0.5Gy of dose due to scatter. 75% of the target volume (brain excluding hippocampus) receives 3Gy (75% of the prescription isodose) and 50% of the target volume receives 100% of the prescription isodose.

A Monte Carlo simulation with smART-Plan was used for treatment planning. The dose volume histogram (DVH) shows that 90% of the hippocampus receives about 0.5Gy assuming zero transmission through the block (Fig 8). Because this dose closely matches the radiochromic film dose (0.52Gy), this suggests that this low dose to the hippocampus is a result of scattered radiation rather than transmission through the block. However, the DVH also shows incomplete coverage of the target volume (brain excluding hippocampus). 75% of the target volume received 3Gy (75% of the prescription isodose) and 50% of the target volume received 100% of the prescription isodose. This was a function of the anatomy of the hippocampus as a paired structure combined with the use of opposed lateral fields. 2D blocks did not allow for direct dose delivery to the target tissue centrally.

### Immunofluorescence for in vivo verification of hippocampal avoidance

**Fig 9:**
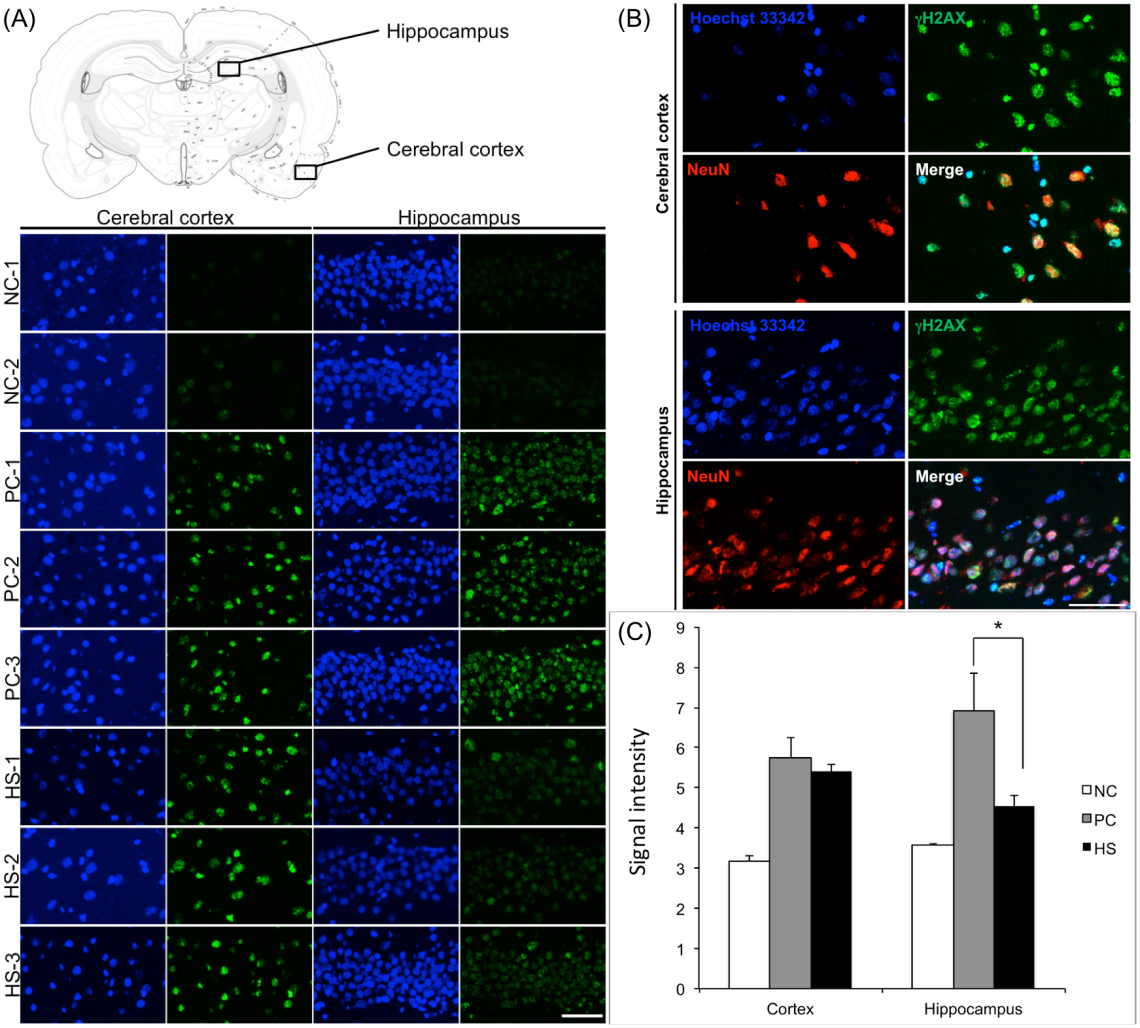
In vivo validation of hippocampal avoidance with radiation in rats treated with brain irradiation with hippocampal avoidance. A. Representative immunofluorescence images of rats treated with no irradiation (Control), whole brain irradiation (WBRT), and hippocampal avoidance brain irradiation (HA). For each rat, a section of brain from the cortex (not spared) and a section from the hippocampus (granule cell layer of the dentate gyrus) is shown. A brain map (top) indicates the locations where the images of the cortex and hippocampus are taken. Scale bar = 250 microns. B. Anti-phospho-Histone H2A.X antibody immunofluorescence signal intensity analysis. The radiation-induced phosphorylation of Histone H2A.X in the hippocampus is significantly decreased when the brain is irradiated with hippocampal avoidance, * P = 0.012 (by student t-test). C. Immunofluorescence images of the cortex and hippocampus of a rat treated with WBRT. Red = NeuN (a neuron-specific marker), Green = phosphorylation of Histone H2A.X. Scale bar = 125 microns.

To verify that the hippocampal avoidance brain irradiation minimized DNA damage to the hippocampus in vivo in rats, we irradiated rats with 4Gy whole brain irradiation (n = 3), 4Gy brain irradiation with hippocampal avoidance (n = 4), and negative control (n = 2). Approximately, 1 hour after irradiation, the rats were killed and the brains were removed for analysis by immunofluorescence to detect phosphorylation of Histone H2A.X, which occurs as a consequence of DNA damage from ionizing radiation. Results for the *in vivo* verification with anti-phospho-Histone H2A.X (reflecting DNA damage) are shown in Fig 9. In the cortex of the rats treated with the hippocampal avoidance protocol, radiation therapy induced a similar large amount of phosphorylation of Histone H2A.X as in rats treated with WBRT (Fig 9A). Quantification of the mean signal intensity of phospho-H2A.X in the cortex of rats treated with the hippocampal avoidance protocol was not significantly different from the signal intensity in the cortex of rats treated with WBRT (5.40 v. 5.75, p = 0.32 in fig 9B). In contrast, in the hippocampus of rats treated with the hippocampal avoidance protocol, radiation therapy induced a markedly decreased amount of phosphorylation of Histone H2A.X compared to rats treated with WBRT. Quantification of the signal intensity of phosphor-H2A.X in the hippocampus of rats treated with the hippocampal sparing protocol was significantly lower than rats treated with WBRT (4.55 v. 6.93, p = 0.012). The signal intensity of 4.55 in the hippocampal avoidance protocol was also modestly higher than in the negative control rats (3.57, p = 0.02)—a difference that was statistically significant.

## Discussion

The technical and clinical challenges encountered in developing a procedure that can accurately treat such a small organ as the rat brain, while sparing the even smaller volume of hippocampal tissue are substantial. This study was undertaken after much preparatory non-clinical work commissioning and evaluating the characteristics and accuracy of the image-guided capabilities of a specialized micro-irradiator device in our lab [9, 10, 11]. Three key challenges were identified: hippocampal localization (achieved using an MRI atlas, with sophisticated registration and deformation capability), treatment planning (including manufacture of tiny lead blocks custom fabricated to match DRR projections, and Monte Carlo dose calculation), and treatment localization (using on-board image-guidance capability of the X-RAD225 irradiator). Working solutions were found for all three challenges and to our knowledge, this is the first time hippocampal avoidance has been successfully performed in rodents. The potential applications for this protocol are broad—allowing for investigations of the indirect and network-dependent effect of radiation on hippocampal stem cells and mature, differentiated neurons which have both been shown to be altered by exposure to radiation [15, 16, 17] Behavioral, electrophysiologic and cellular experiments performed under hippocampal-avoidance conditions would then have direct parallels with RTOG 9903 [7].

Despite the benefits of our new model system for studying hippocampal avoidance in rats, there are some limitations to our study. First, a small number of rats (n = 6) were irradiated. Nonetheless, this samples size provided sufficient statistical power to detect a robust difference between the DNA damage as measured by phosphorylated H2A.X in the rats treated with the HA protocol and the rats treated with WBRT. Also, even in rats treated with the hippocampal-avoidance protocol, we did observe increased DNA damage in the hippocampus of these rats when compared to unirradiated controls. This likely indicates that the blocks shielded 87% of the dose from the hippocampus, but 13% of the radiation dose (approximately 0.5 Gy) still reached the hippocampus. Nonetheless, this compares favorably with the RTOG 0933 protocol specifications in which a mean dose of 9Gy is allowed to the hippocampal avoidance structure with a prescription dose of 30Gy to the target volume (70% dose reduction). Additionally,, coverage of the target volume (brain minus hippocampus) was suboptimal due to the opposed lateral field technique resulting in underdoing of some of the diecephalon centrally. However, modeling the cortical changes mediated by radiotherapy with a subsequent circuit effect on the hippocampus while adequately sparing the hippocampus is likely most relevant for a rodent model. Lastly, it is important to note that our treatments were planned and delivered to healthy, wild-type Wistar rats for the purpose of investigating normal tissue toxicity. These procedures will likely need adapting for application to rat models with intracranial tumors, where brain anatomy and segmental volumes may change relative to the atlas. However, the use of deformable image registration techniques may enablethis application.

Additionally, we have only utilized the HA protocol for a single fraction thus far. For multi-fraction experiments, It may be an advantage to incorporate head immobilization to minimize intra-fraction motion. Alternatively, daily CBCT scans can be acquired for accurate daily set-up for multi-fraction experiments. Despite these limitations, this work demonstrates that it is feasible to use complex treatment planning to deliver conformal brain irradiation that spares the hippocampus in a rat. We anticipate that this HA sparing protocol will be useful for future studies to understand the consequences of irradiation to the hippocampus at the network-level. Moreover, the semi-deformable registration of the brain MRI atlas and the CBCT could be applied for new treatment plans to minimize radiation dose to other brain structures in the future.

## Supporting Information

S1 Fig Registration points F1-6 on MRI and CBCT images. (PDF)

S1 Video Registrations points F1-6 on 3D skull rendering. (AVI)
