# Supplementary material for: Treatment Planning and Delivery of Whole Brain Irradiation with Hippocampal Avoidance in Rats
Source: PLoS One. 2015 Dec 4;10(12):e0143208. doi: 10.1371/journal.pone.0143208 (PMC4670078; doi:10.1371/journal.pone.0143208)
Supplement: S1 Fig — (PDF) [file pone.0143208.s001.pdf]

Supplemental figure 1: Registration points F1-6 on MRI and CBCT images

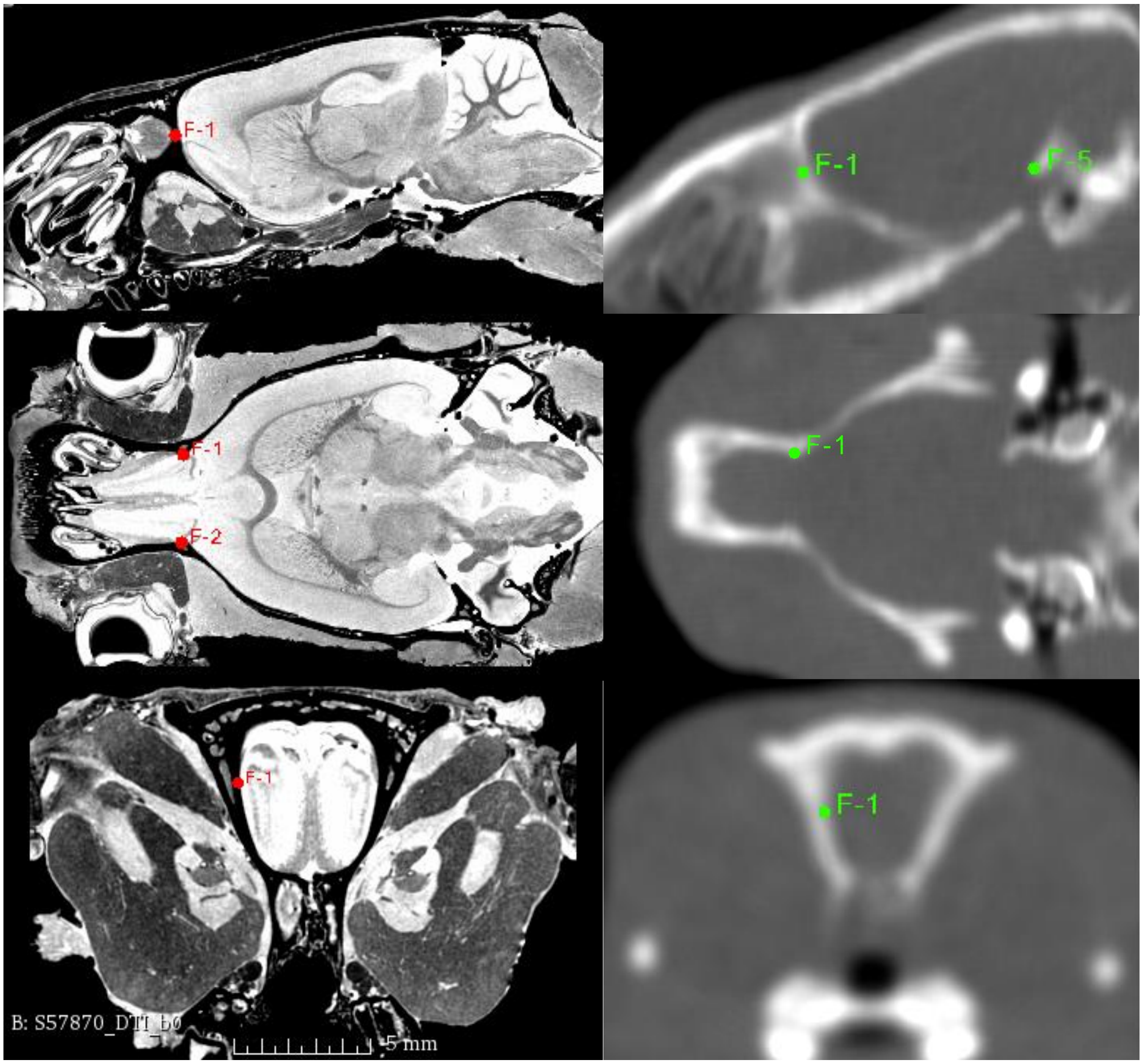

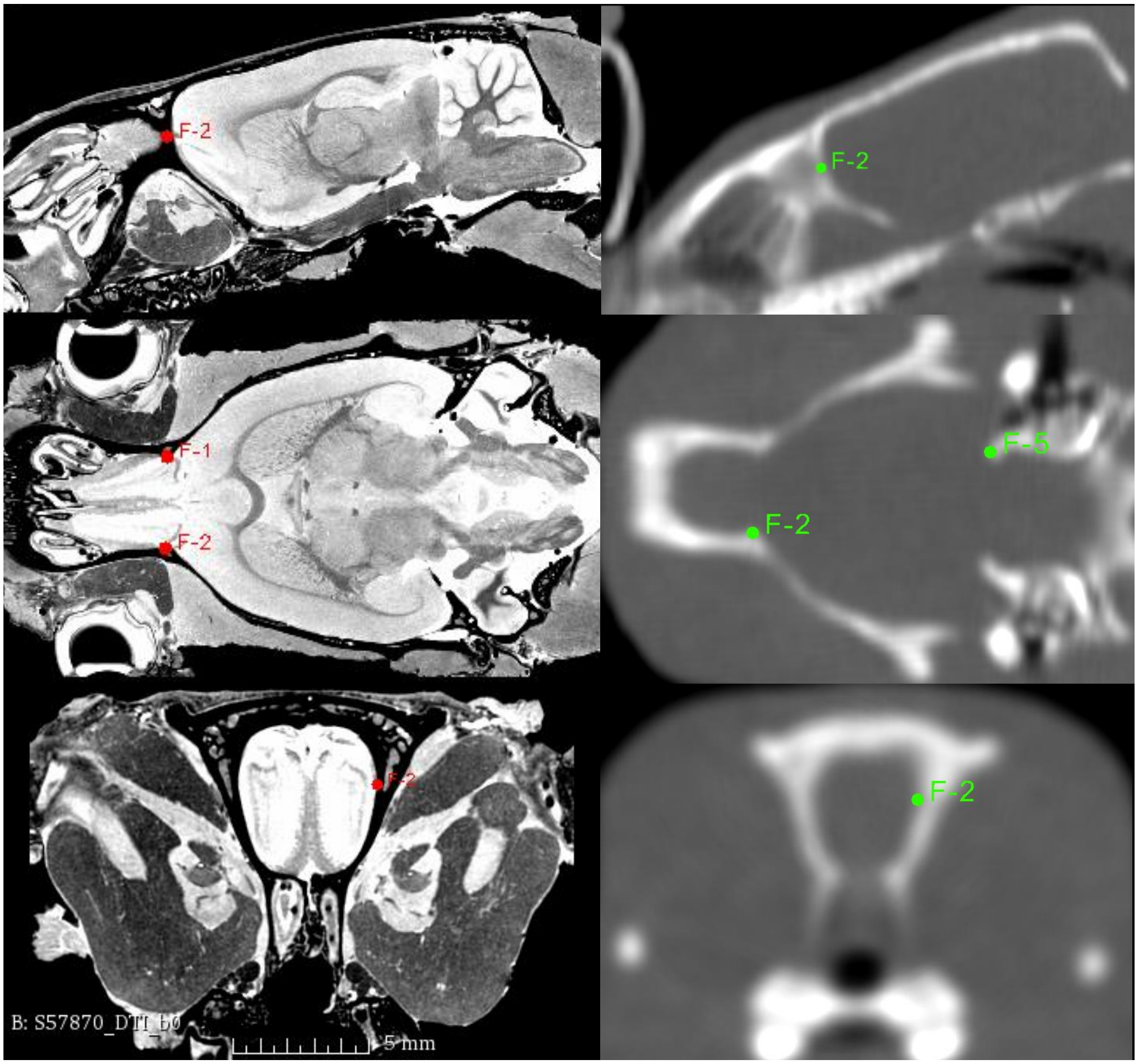
F-2
F-2
F-1
F-2
F-5
F-2
F-2
F-2
B: S57870_DTI_b0
5 mm

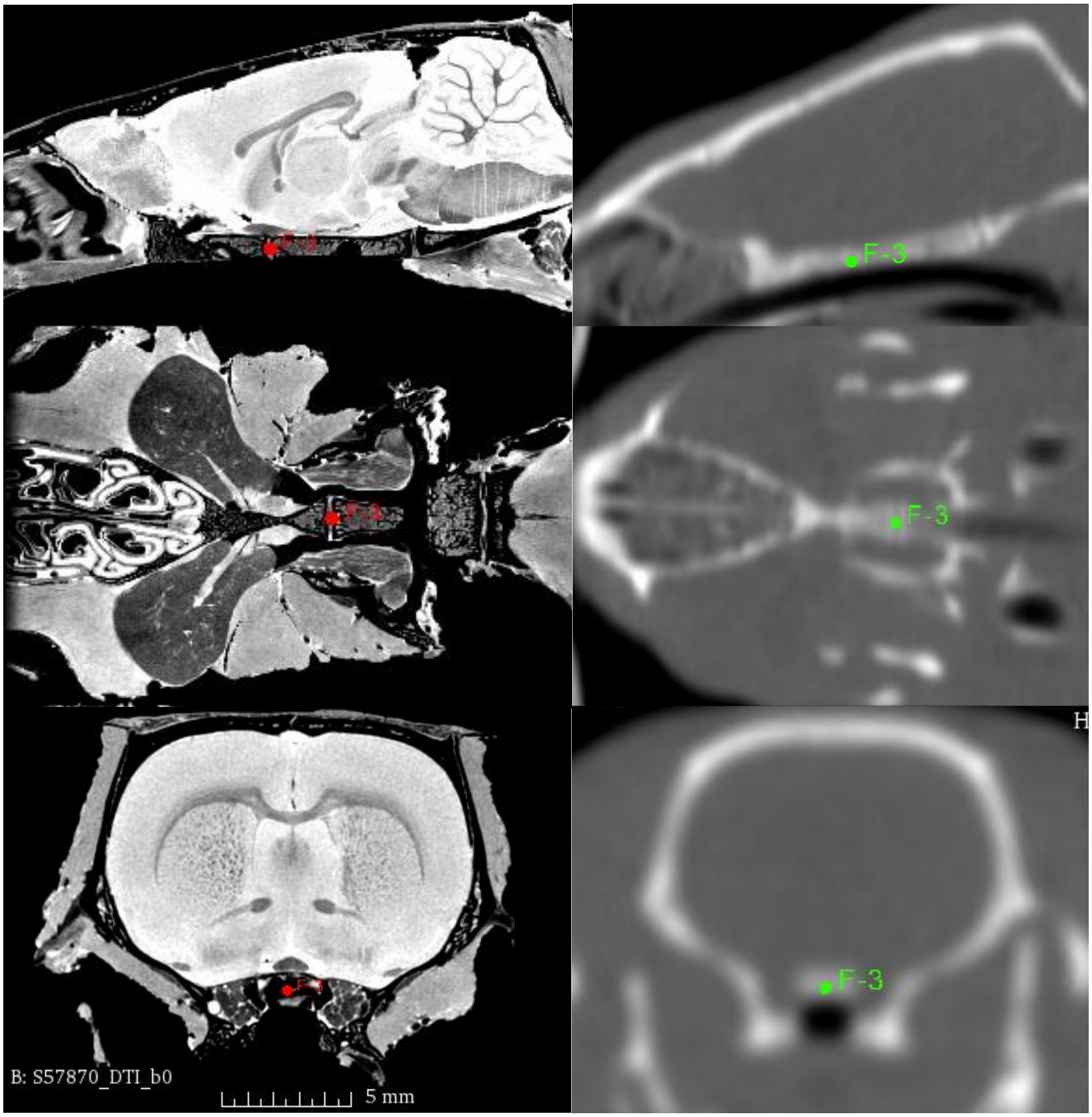
F-3
F-3
F-3
F-3
F-3
F-3
H
B: S57870_DTI_b0
5 mm

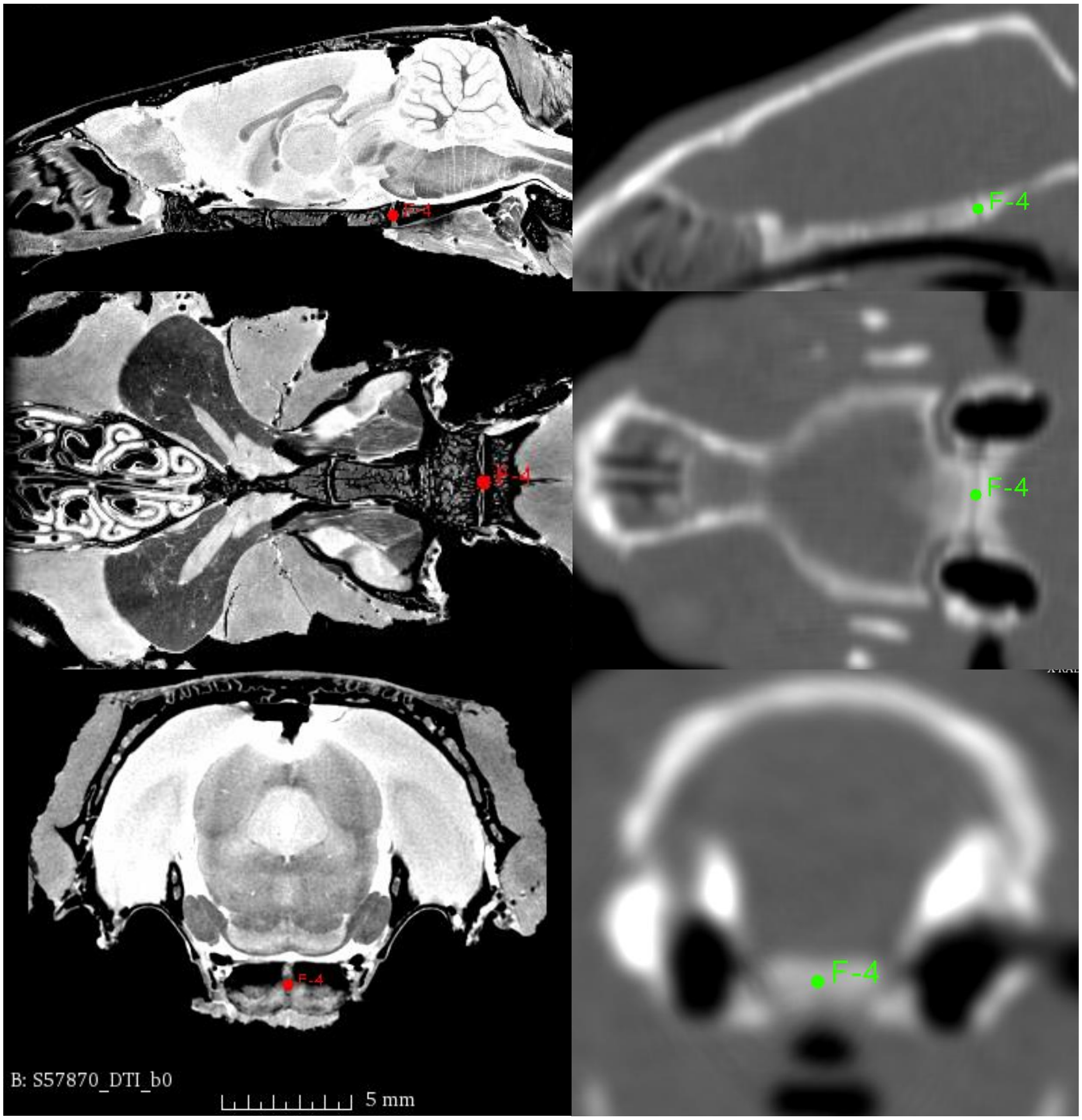
F-4
F-4
F-4
F-4
F-4
F-4
B: S57870_DTI_b0
5 mm

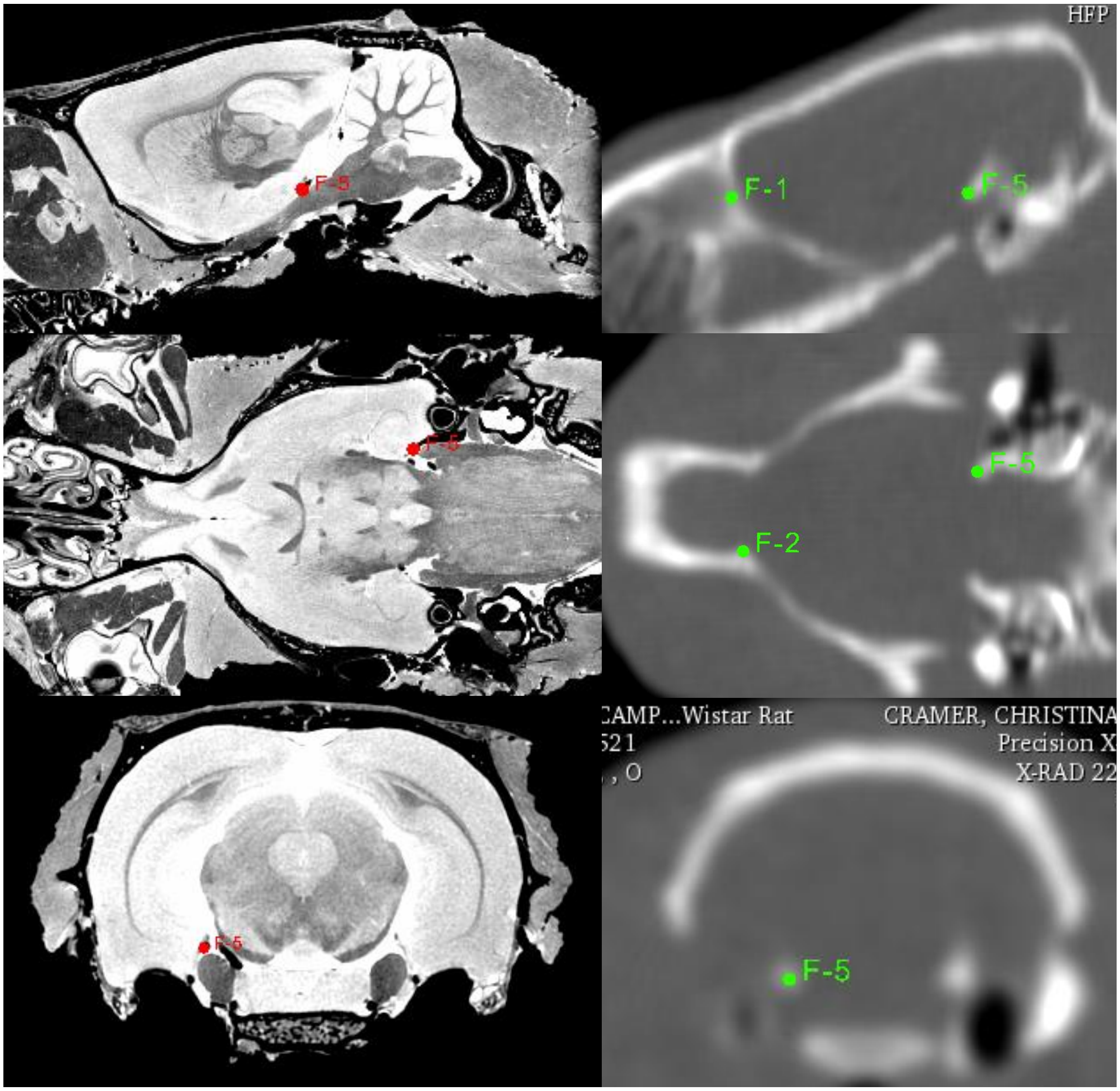
HFP
F-5
F-1
F-5
F-5
F-5
F-2
CAMP...Wistar Rat
CRAMER, CHRISTINA
521
Precision X
, O
X-RAD 22
F-5
F-5

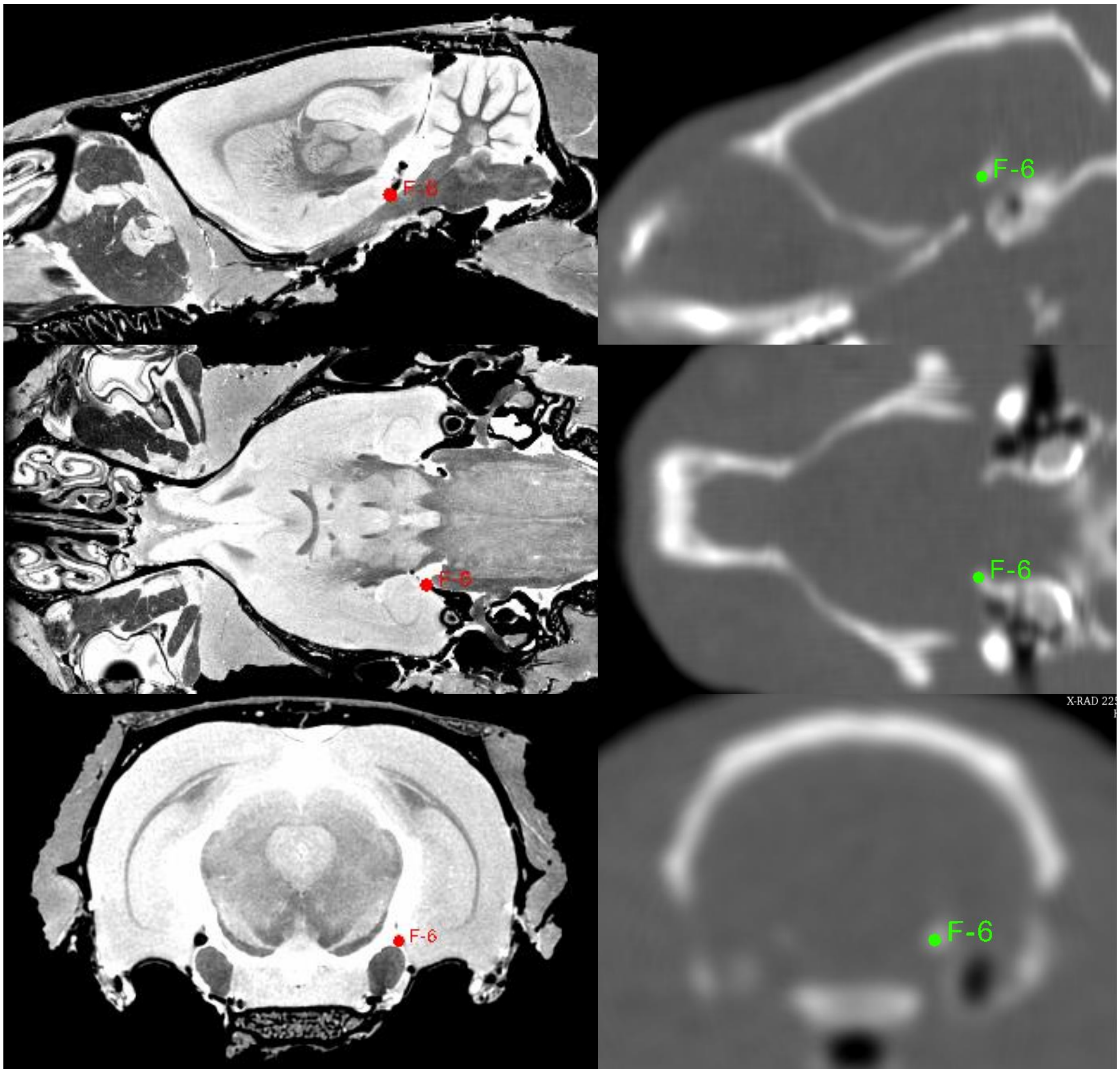
F-6
F-6
F-6
F-6
F-6
F-6
